# Three-Year Cereal: Field Bean Intercropping Greatly Reduced Weed Abundance with Small Changes in Functional Composition

**DOI:** 10.3390/biology15030239

**Published:** 2026-01-28

**Authors:** Iduna Arduini, Dayana Naimid Esnarriaga, Marco Mariotti, Sergio Saia, Francesco Giovanni Salvo Angeletti, Silvia Pampana

**Affiliations:** 1 Department of Agriculture, Food and Environment, University of Pisa, Via del Borghetto, 80, 56124 Pisa, Italy; silvia.pampana@unipi.it; 2 Forschungsinstitut für Biologischen Landbau—FiBL, Ackerstrasse 113, 5070 Frick, Switzerland; naimid.esnarriaga@fibl.org; 3 Department of Veterinary Science, University of Pisa, Viale delle Piagge, 2, 56124 Pisa, Italy; marco.mariotti@unipi.it (M.M.); sergio.saia@unipi.it (S.S.); francesco.angeletti@phd.unipi.it (F.G.S.A.)

**Keywords:** average frequency, barley, cosmopolitan species, NP fertilization, triticale, weed biomass, tall weeds, type contribution, weed richness

## Abstract

Weeds reduce crop yield but may also support agroecosystem biodiversity. The biomass and functional composition of weed communities were evaluated in cereal–field bean intercrops and sole crops, with and without NP fertilization. Intercrops were repeatedly grown in the same plots using 1:1 and 2:1 cereal:field bean row ratios, while sole crops were sown at low or high density and rotated. Weeds were sampled at cereal flowering and maturity stages. Fertilization had little effect on weed biomass but tended to reduce species richness and favor cosmopolitan species. Intercropping reduced weed biomass two- to sevenfold compared with high-density cereal and legume sole crops. Plot richness was 39% lower in intercrops than in field bean and low-density cereal crops. Over three years, weed functional composition was influenced more by year than by crop system or fertilization. However, the contribution of tall weeds increased in intercrops: 51% compared to 42% in high-density sole crops and approximately 31% in low-density ones. The average frequency of types indicated early shifts in weed community structure. In conclusion, continuous cereal–field bean intercropping controlled weeds more effectively than rotated sole crops, with a slight decline in species richness and minor shifts in functional composition.

## Simple Summary

In sustainable crop production, weed management presents special challenges. On the one hand, uncontrolled weed growth can hinder the achievement of quantitative and qualitative standards that meet market and societal demands. On the other hand, the positive contribution of weeds to the conservation of species richness and diversity within agroecosystems is a growing public concern. To address this issue, the influence of cropping system and mineral fertilization on the biomass and functional composition of weed communities was investigated in a three-year experiment in which field bean and winter cereals, either triticale or barley, were grown as intercrops and sole crops, with and without nitrogen and phosphorus fertilization. The simultaneous growth of field beans and cereals in the same field proved to reduce weed growth more effectively than sole crops, irrespective of mineral fertilization. Intercropping did not markedly change the functional composition of weed communities but slightly reduced weed richness and favored tall weeds. Fertilization also tended to reduce species richness and favored widespread weeds at the expense of narrowly distributed species. Intercropping could therefore represent an effective alternative to chemical weed control, although its potential long-term adverse effects on weed biodiversity should be monitored.

## 1. Introduction

Since the origin of agriculture, weeds have posed challenges by competing with crops for light, water, and nutrients and reducing crop yield and quality [1]. In some circumstances, weeds may also host symbiotic fungi, insect pests, and diseases, posing health hazards to humans and livestock [2,3]. Weed management requires significant labor, mechanical operations, and herbicide applications, making them a costly constraint to crop production, both in the traditional cropping systems of developing countries [4,5] and in organic agriculture [6,7]. In intensive systems, however, the past massive use of herbicides has negatively affected the environment and promoted weed resistance [8]. In recent decades, increasing attention has been paid to the positive role of weeds in supporting biodiversity and ecosystem services [9,10]. For example, widespread weeds such as *Cirsium* and *Rumex* can provide more nectar to pollinators than recommended flower mixtures [11]. Thus, sustainable cropping systems aim to balance weed control to maintain crop yield while preserving ecosystem health [12].

Increasing crop competition is a key strategy for biological weed control and can be achieved by selecting vigorous genotypes and increasing crop density [6,13]. To avoid intra-crop competition, high plant density is obtained by intercropping, defined as the simultaneous cultivation of two crop species in the same field [9,14]. Weed suppression in intercrops relies mainly on early competition for light and for water and nutrients, particularly when complementary crops like N_2_-fixing legumes and cereals are combined [15,16,17]. Previous studies reported that weed biomass in intercrops is often intermediate to, or lower than, that of component sole crops [18,19]. Other authors suggested that weed suppression depends more on crop biomass than on crop diversity [20], although intercrops may provide more stable weed control across environments [21]. Recent studies pointed out that species composition influenced the weed suppressive ability of intercrops more than mixing ratio, planting density, and spatial arrangement [22,23].

Intercropping cereals with legumes is particularly relevant for weed management, as legume sole crops generally favor weed growth due to low sowing density, slow early growth, and limited competitiveness for mineral nitrogen [24,25]. In addition, biological N_2_ fixation may increase soil nitrogen availability, stimulating weed growth also in subsequent cereal crops [26,27,28]. Reduced weed growth in cereal–legume intercrops has been largely attributed to the high early nitrogen uptake of cereals, which was also observed at low cereal proportions and low nitrogen availability [14,29,30].

Weed biomass and species richness are not necessarily correlated, although both are important from an agroecological perspective [31]. Most intercropping studies, however, have primarily focused on weed biomass, paying comparatively less attention to changes in species composition. Because weed assemblages are strongly influenced by site conditions and cropping history, functional trait-based approaches provide a robust framework for comparing weed communities across cropping systems [32,33]. Weed species commonly have traits, such as short life cycles and persistent soil seed banks [1,34], and management methods may selectively favor specific functional groups. For example, conservation agriculture has been shown to promote persistent weeds and perennial monocots [35,36]. Likewise, increased crop competition achieved through intercropping or high cereal sowing densities reduces the biomass of later-emerging weeds but may also lead to the dominance of a few highly competitive species [1,13,31,37].

We hypothesized that continuous cereal–legume intercropping and rotated sole crops would differently affect weed biomass and functional composition, independently of initial community structure.

To test this hypothesis, we evaluated weed biomass, species richness, and functional traits over three consecutive years in cereal–field bean intercrops and sole crops. All crops were grown at different densities and component ratios, with or without mineral nitrogen and phosphorus fertilization, in two adjacent fields differing only in recent cropping history. The growth and structure of the weed communities were estimated in terms of overall biomass and in the partitioning of biomass within grasses, forbs, and legumes. The weed communities were also analyzed in terms of richness and frequency of plant functional types. By combining a multi-year field experiment with a trait-based analysis across crop densities, intercrop ratios, fertilization regimes, and contrasting field histories, our study introduces a novel framework to elucidate how intercropping and sole cropping shape weed community assembly beyond simple suppression effects, thereby integrating agronomic and ecological perspectives in weed research.

## 2. Materials and Methods

### 2.1. Experimental Site and Design

A large-scale field comparison was carried out at the experimental station “Rottaia” of the Department of Agriculture, Food and Environment (University of Pisa, Italy) over three consecutive cropping seasons (2017–18 (year I), 2018–19 (year II), and 2019–20 (year III)) to assess weed biomass and functional composition in continuous cereal–field bean intercrops compared with rotated sole crops.

The experimental station is located approximately 3 km from the sea (43°40′34″ N, 10°18′41″ E) and 0 m a.s.l. The climate is hot-summer Mediterranean, with mean annual maximum and minimum daily air temperatures of 20.2 and 9.5 °C respectively, and mean rainfall of 971 mm per year.

Each year, the experimental design consisted of two fertilizer treatments and six crop systems, arranged in a split-plot experimental design, with fertilizer treatments as main plots and crop systems as subplots, with two replicates. Plots measured 6 m × 15 m. This design was replicated in two fields (A and B) located approximately 300 m apart but differing in recent cropping history: in field A, the preceding crop was low-tillage oat, while field B had not been cultivated for two years and had previously been covered by a legume mixture. Thus, each year, the complete design was a split-split plot with fields as the main plots, fertilizer treatments as subplots, and crop systems as sub-subplots, with two replicates. Fertilizer treatments were no N and P supply (0) and combined N and P fertilization (NP). Crop systems included two cereal-legume intercrops (IC) differing in cereal:legume row ratio (1:1 IC and 2:1 IC) and one high-density (C100 and Fb100) and one low-density (C60 and Fb60) sole crop (SC) for both the cereal and the legume (Table 1). The main soil properties of the two fields at the beginning of the research are reported in Table 2.

Sole crops were sown at low and high plant density, mimicking the seed densities in the intercrops. The cereal crop was triticale (*Triticosecale* Witt. cv. ‘Trismart’) in year I and a six-row barley hybrid (*Hordeum hexastichon* L. cv. ‘Jallon’) in years II and III; this replacement was necessary to better match the phenological development of the companion crops. Based on the different cultural techniques of triticale and barley, the seed density of cereal sole crops and, consequently, the proportion of seeds in the intercrops differed in year I and years II-III (Table 1). Field bean (*Vicia faba* minor Beck cv. ‘Vesuvio’) was used as the legume crop throughout the experiment. From one cropping season to the next, IC was grown in the same plots, whereas SC were rotated with each other.

**Figure 1:**
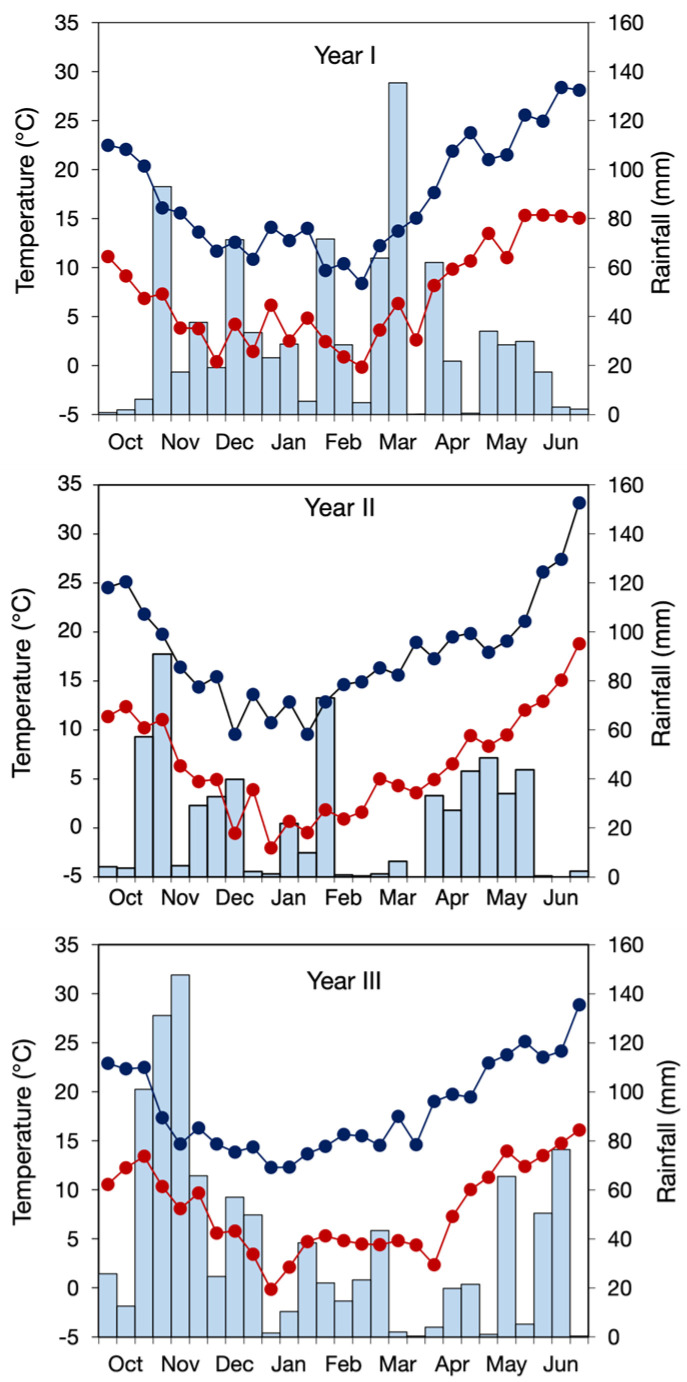
Air minimum (red line) and maximum (blue line) temperatures and rainfall (bars) for the period October–June of year I (2017–18), year II (2018–19), and year III (2019–20). Temperatures are 10-day averages, and rainfall is 10-day sums.

Meteorological data were obtained from a weather station located close to the trial site. Temperatures from October to June did not vary greatly among cropping seasons, with mean values ranging from 12.2 to 13.2 °C, minimum values from 6.6 to 8 °C, and maximum values from 17.4 to 18.4 °C (Figure 1). In contrast, cumulative rainfall from October to June differed substantially, amounting to 842, 612, and 1015 mm in years I, II, and III, respectively.

Weed control was not performed throughout the entire research to avoid interference with treatment effects on weed selection. Fertilizer application each year consisted of 100 kg K_2_O ha^−1^ in all plots and, in NP plots, 120 kg N ha^−1^ plus 100 kg P_2_O_5_ ha^−1^. Phosphorus and potassium were distributed before ploughing, whereas nitrogen was split into three applications: 30 kg N ha^−1^, as ammonium sulphate at pre-sowing, followed by two equal applications of 45 kg N ha^−1^ as urea when the high-density cereal sole crop (C100) reached the pseudostem erection stage (BBCH 30), according to the scale of growth stages of the Federal Biological Research Centre for Agriculture and Forestry [38], and 15 days later. Nitrogen fertilization was also applied to field bean to maintain uniform treatments [39].

### 2.2. Weed Harvest and Measurements

Sowing and harvest dates are reported in Table 3. In year I, weeds were harvested at the vegetative (BBCH 30), flowering (BBCH 69), and maturity stages (BBCH 89) of triticale; in years II and III, at BBCH stages 69 and 89 of barley. At each harvest, weeds and crops were manually cut at ground level in two 0.5 m × 0.5 m sampling units per subplot (fertilizer × crop system). Plants were separated into crops and weeds; weeds were further separated into grasses (Gr), forbs (Fo), and legumes (Lg) and oven-dried at 65 °C to constant weight.

The weed communities of fields A and B were characterized in terms of species richness, frequency, and similarity. Weeds were identified following Pignatti [40,41,42], and scientific names were updated according to the Portal to the Flora of Italy 2025.1 (https://dryades.units.it/floritaly/index.php; accessed on 5 December 2025) [43].

Richness was estimated as the total number of species recorded across all subplots of one field over the three cropping seasons. Species frequency (*SF*) was calculated separately for each year and field as:

*SF* = *Qis*/*Q* × 100

where *Qis* is the number of sampling units in which a given species occurred, irrespective of the number of individuals, and *Q* is the total number of sampling units analyzed per year and field (48 = 6 crop systems × 2 fertilizer levels × 2 harvests × 2 sampling units).

Similarity between weed communities was assessed using the Sørensen similarity index (*IS*), calculated as *IS* = 2*J*/(*a* + *b*) × 100 [44], where *a* and *b* are the numbers of species of the two weed communities and *J* the number of shared species. The index ranges from 0 (completely different) to 100 (identical).

### 2.3. Analysis of Richness

Mean richness (mR), defined as the number of weed species per 0.5 m × 0.5 m sampling unit, was calculated to evaluate the impact of NP fertilizer and crop systems on weed diversity, accounting for field composition and yearly climate variation.

Plot richness (pR) was calculated as the total number of species recorded in the four sampling units collected at either flowering or maturity within each subplot in one year.

### 2.4. Analysis of Functional Categories

To determine if NP fertilization and different crop systems had varying effects on weed functional traits, species were classified into functional groups based on expected responses to fertilization and cropping system, as supported by previous research [1,13,21,35,36,37]. These groups included life span (A for annuals, B for biennials, P for perennials, and AP for species that can be both annual and perennial), flowering time compared to crops (E for early, S for spring, and L for late), plant size relative to crops (s for small, m for medium, and t for tall), and geographic distribution range (N for narrow and W for wide) (see Table A1). Raunkiaer’s life forms (T, therophyte; H, hemicryptophyte, G, geophyte; T-H, intermediate forms) were also included because of their widespread use in assessing plant community responses to disturbance. Species functional classification (Table A2) followed Pignatti [40,41,42]; field observations of flowering time and relative size to crops were used as the ground truth when in conflict with Pignatti.

The influence of NP fertilization and crop system on weed community structure was analyzed by calculating the relative contribution of each type (*TC*) within functional categories, based on the Daget and Poissonet index [45]:

$$TC=\frac{T{\Sigma a}_{i}}{\sum T\Sigma a_{i}}\times 100$$

where *T*Σ*a*_i_ represents the number of sampling units in which species *i* belonging to type *a* occurred in a plot, irrespective of individual abundance. As TC depends on both species richness and frequency, relative richness (RR) and average frequency (AF) were also calculated. Relative richness was defined as the proportion of species belonging to a given type relative to the total number of species recorded annually in a subplot, while AF was calculated as the mean frequency of all species assigned to a given type within a subplot each year.

### 2.5. Statistical Analysis

Due to the use of different cereal species (triticale in year I, barley in years II and III), variations in cropping systems—since year I did not have the same preceding intercrop or rotation history as the following years—and differences in variances, the biomass data were analyzed separately for year I and for years II and III. Analyses were conducted using ANOVA with the GLIMMIX procedure of SAS/STAT 9.4 version M8, which allows modelling of non-normal data and correction for heteroscedasticity [46]. In year I, harvest stage was treated as a time factor, field as the main plot, fertilizer level as the subplot, and crop system as the sub-subplot, with two replicates. In years II and III, year was considered the main plot, harvest stage the time factor, field the subplot, fertilizer level the sub-subplot, and crop system the sub-sub-subplot, with two replicates. Year was treated as a random effect, while harvest stage, field, fertilizer level, and crop system were treated as fixed effects. To fulfill assumptions of normality and homogeneity of variance, data were transformed. Weed biomass data were log(x + 1) transformed because of the multiplicative effect due to growth and the great differences due to treatments, and forb proportion data were arcsine transformed because there were several proportions close to 0 and/or close to 1. After transformation, the inspection of residuals indicated approximate normality (W = 0.966 for weed biomass and 0.86 for forb proportion) and homoscedasticity. Therefore, transformed data were analyzed using ANOVA. Post hoc comparisons were performed using Tukey’s test, with significant differences declared at α = 0.05 [47].

Mean species richness was analyzed by pooling data from all years, with harvest stages treated as replicates under the assumption that the weed community structure did not change after flowering. Year was considered the main plot, field the subplot, fertilizer level the sub-subplot, and cropping system the sub-sub-subplot, with four replicates. Species counts were square root transformed (*x* + 0.5) prior to analysis.

To assess the effects of crop system, NP fertilization, and cropping season on weed community structure, a canonical discriminant analysis (CDA) was performed, including mean richness per sampling unit (mR), relative contribution of types (TC), average frequency of types (AF), and relative richness of types (RR), resulting in 60 variables (Supplementary Files S1–S7b). The field and harvest stages were treated as random samples within treatments (Supplementary Files S2, S6b, S7b). Correlation analysis reduced the dataset to 23 variables (Supplementary File S3), but two showed linear dependence, resulting in a singular covariance matrix. Applying a threshold of |r| < 0.65, 21 variables were retained (Supplementary File S4), standardized (mean = 0, SD = 1), and analyzed using the CANDISC procedure in SAS/STAT 9.4. Mahalanobis distance (*p* < 0.05) was used to assess treatment separation (Supplementary File S5) [48]. Variables highly correlated with the first three canonical axes (*p* < 0.05) were retained. These variables were also tested by means of ANOVA for differences in response to NP fertilization, crop system, and their interaction.

## 3. Results

### 3.1. Composition of Weed Communities

Throughout the three-year field experiment, 35 species were observed in field A and 38 in field B, for a total of 48 weed species (Table 4 and Table 5). Of these, two were identified only at the genus level, and one could be assigned only to forbs, with no species-level identification. Forbs (33 species) were the most represented functional group, followed by grasses and legumes, with nine and six species, respectively.

Weed communities did not differ greatly between the two fields, despite differences in preceding crops, as the Sørensen index revealed an overall field similarity of 68.5%. When years were analyzed separately, field similarity increased progressively over time, from 58.6% in year I to 61.5% in year II and 66.7% in year III. A total of 25 species were common to both fields, while 10 species were exclusive to field A and 13 to field B. Nine species, *Equisetum arvense*, *Lolium multiflorum*, *Poa trivialis*, *Helminthotheca echoides*, *Sonchus arvensis*, *Papaver rhoeas*, *Convolvulus arvensis*, *Polygonum aviculare,* and *Anagallis arvensis*, were observed in all years and in both fields (Table 4), with *L. multiflorum* and *H. echioides* showing the highest frequencies. The proportion of grasses was similar between fields, whereas legumes were more represented in field B than in field A (Table 5). The weed community in field B also included a slightly higher proportion of annual, widely distributed cosmopolitan, and subcosmopolitan species. Year-to-year variability in the number of observed species ranged from 16 to 33 in field A and from 23 to 27 in B (Table 4), and, in both fields, richness was lowest in year II. Forbs tended to increase their frequency in time, while the opposite occurred for grasses and legumes (Table 4). Because of the low and declining frequency of legume weeds, they were included in forbs for further analysis.

### 3.2. Weed Biomass

#### 3.2.1. Year I

Results of the ANOVA indicated that weed biomass changed significantly in response to the interactions Harvest × Field, Field × Crop System, and Harvest × Fertiliser × Crop System. Further details of the ANOVA and Tukey’s test are reported as Supplementary Material (Table S1).

**Figure 2:**
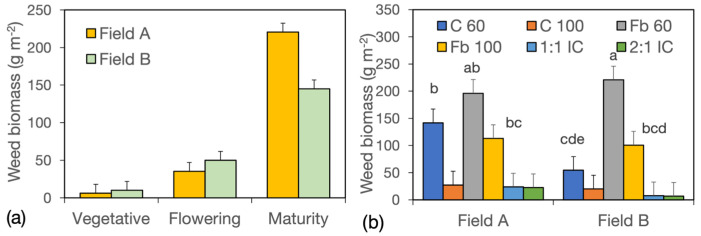
Weed biomass in year I. (**a**) Interaction harvest stage × field; data are means of two fertilizer levels, six crop systems, and two replicates (*n* = 24). (**b**) Interaction field × crop system; data are means of two harvest stages, two fertilizer levels, and two replicates (*n* = 8). Error bars denote HSD (*p* ≤ 0.05, Tukey’s test). Different letters above columns denote significant differences; unlabelled columns are ‘d’.

Averaged across fertilizer and crop system treatments, weed biomass increased from the vegetative harvest (8.2 g m^−2^) to the flowering harvest (42.6 g m^−2^), with no differences between fields (Figure 2a). After flowering, weed biomass increased sixfold in field A and threefold in field B, reaching 221 g m^−2^ in the former and 145 g m^−2^ in the latter at maturity. Weed biomass varied markedly among crop systems, with the highest values recorded in the low-density field bean (Fb60; 208 g m^−2^), followed by the high-density field bean (Fb100) and the low-density cereal (C60), with 107 and 98 g m^−2^, respectively (Figure 2b). Much lower weed biomass was observed in the high-density cereal (C100; 24 g m^−2^) and in the two intercrops, with 16 g m^−2^ in the 1:1 IC and 15 g m^−2^ in the 2:1 IC. Differences between fields were detected only for C60.

**Figure 3:**
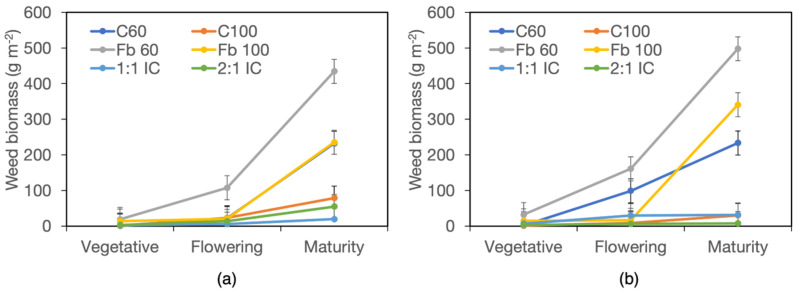
Weed biomass in year I. Interaction harvest stage × fertilizer × crop system. (**a**), 0 fertilizer; (**b**) NP fertilizer. Data are means of two fields and two replicates (*n* = 4). Error bars denote HSD (*p* ≤ 0.05, Tukey’s test).

At the vegetative harvest, weed biomass was significantly higher in the fertilized low-density field bean (Fb60) than in the fertilized and unfertilized cereal crops and intercrops (31.7 vs. 2.5 g m^−2^), while intermediate values were recorded in the other field bean sole crops (15.7 g m^−2^) (Figure 3a,b). Differences in weed biomass among crop systems increased as the cropping cycle progressed. At flowering, the highest weed biomass was still observed in Fb60 under both fertilizer treatments, but a marked increase was also detected in C60 with NP fertilization. At maturity, weed biomass ranked as Fb60 > Fb100 = C60 > C100 = ICs in the unfertilized treatment (Figure 3a) and as Fb60 > Fb100 > C60 > C100 = ICs in the NP treatment (Figure 3b).

The response to fertilization was generally weak. However, NP tended to increase weed biomass at flowering in the low-density crops C60 and Fb60 and at maturity in Fb100 and the 1:1 IC, while slightly decreasing it in C100 and the 2:1 IC. On average, weed biomass at maturity was approximately 28 g m^−2^ in intercrops, about double in C100 (54 g m^−2^), ninefold higher in C60 and Fb100 (260 g m^−2^), and sixteenfold higher in Fb60 (466 g m^−2^).

**Figure 4:**
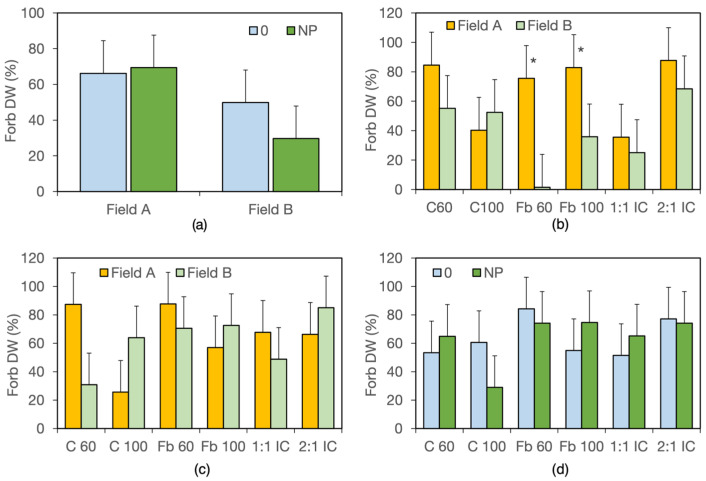
Proportion of forbs (%) in the weed biomass. (**a**) Interaction field × fertilizer in year I; data are means of two harvest stages, six crop systems, and two replicates (*n* = 24). (**b**) Interaction field × crop system in year I; data are means of two harvest stages, two fertilizer levels, and two replicates (*n* = 8). (**c**) Interaction field × crop system in years II and III; data are means of two years, two harvest stages, two fertilizer levels, six crop systems, and two replicates (*n* = 72). (**d**) Interaction fertilizer × crop system in years II and III; data are means of two years, two harvest stages, two fields, and two replicates (*n* = 16). Error bars denote HSD (*p* ≤ 0.05, Tukey’s test). * denotes significant differences.

The proportion of forbs within total weed biomass increased from 45.7% to 61.8% between flowering and maturity, averaged across fields, fertilizer levels, and crop systems. Significant Field × Fertiliser and Field × Crop System interactions were also detected (Table S2). In field A, forbs accounted for approximately 68% of weed biomass irrespective of fertilization, whereas in field B they represented about 50% without fertilization and only 30% with NP application (Figure 4a). The proportion of forbs varied widely among fields and crop systems, but only in field bean sole crops were values significantly higher in field A than in field B (Figure 4b).

#### 3.2.2. Years II and III

**Figure 5:**
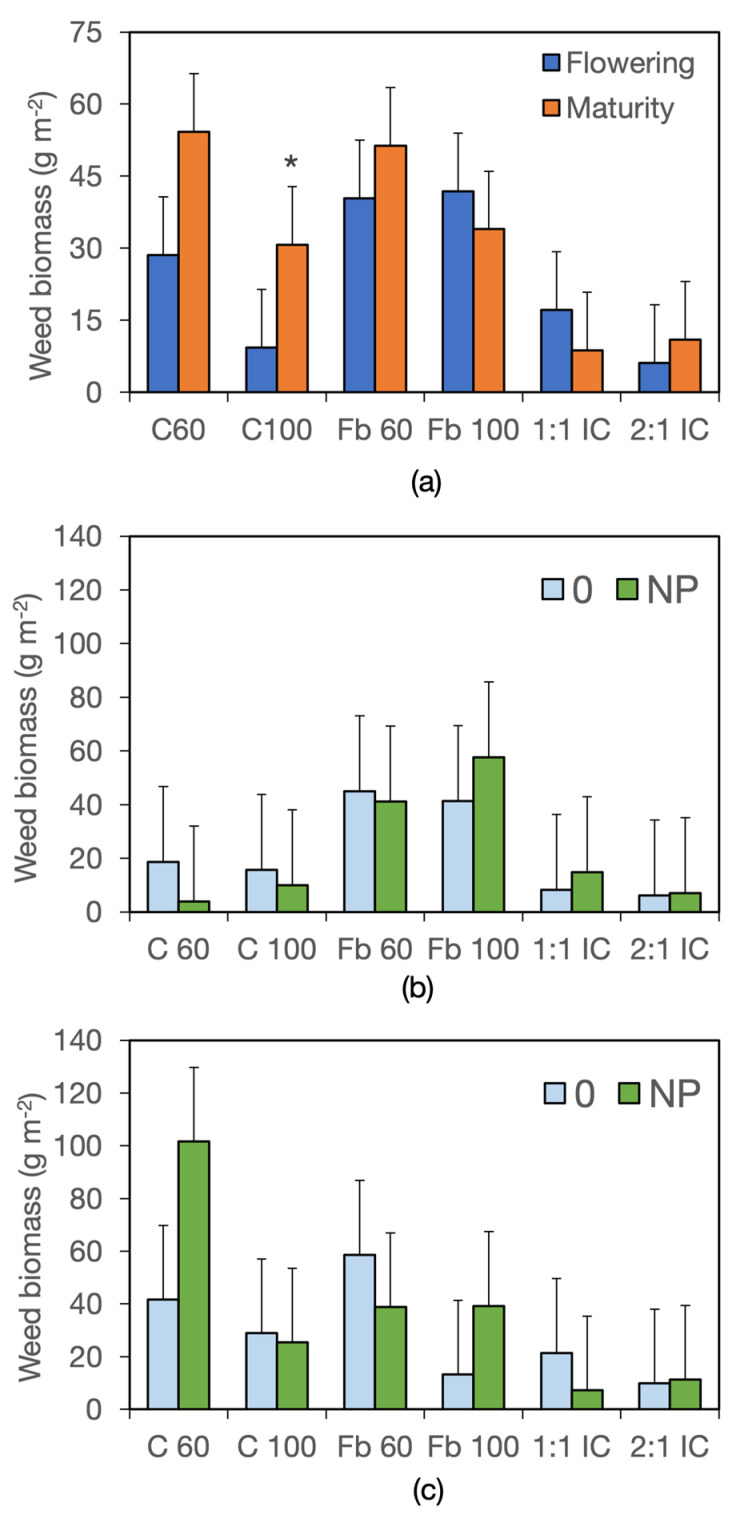
Weed biomass in years II and III. (**a**) Interaction harvest stage × crop system; data are means of two years, two fields, two fertilizer levels, and two replicates (*n* = 16). (**b**,**c**) Interaction field × fertilizer × crop system. (**b**), field A; (**c**), field B; data are means of two years, two harvest stages, and two replicates (*n* = 8). Error bars denote HSD (*p* ≤ 0.05, Tukey’s test). * denotes significant differences.

In the following years, weed biomass was overall much lower, reaching approximately 21.6 g m^−2^ in year II and 33.9 g m^−2^ in year III. Averaged across years, harvests, and fertilizer and crop system treatments, weed biomass differed between fields, amounting to 22.4 g m^−2^ in field A and 33.1 g m^−2^ in field B. The significant Harvest × Crop System interaction indicated that weed biomass increased markedly between harvests only in cereal sole crops, doubling in C60 and tripling in C100 (Figure 5a, Table S3). Moreover, a significant Field × Fertiliser × Crop System interaction was detected, highlighting that weed growth was more variable in sole crops than in intercrops in response to field and fertilizer treatments, with the greatest differences observed in the low-density cereal C60 (Figure 5a,b).

Similarly to year I, weed biomass was substantially lower in intercrops than in sole crops, with average values of 8.6 g m^−2^ in the 2:1 IC and 12.9 g m^−2^ in the 1:1 IC, compared with values ranging from 20 g m^−2^ in C100 to 45.9 g m^−2^ in Fb60 in sole crops. A positive response to fertilization was detected in Fb100 and C60 only in field B.

The proportion of forbs within total weed biomass averaged 56% in year II and 71% in year III and did not differ between harvests, being 61% at flowering and 67% at maturity when averaged across years and treatments (Table S4). ANOVA detected significant Field × Crop System and Fertiliser × Crop System interactions for the proportion of forbs within weed biomass. Differences between fields were greatest in both cereal sole crops (Figure 4c), and C100 showed the strongest response to fertilizer addition (Figure 4d). However, for no crop system did the proportion of forbs differ significantly in response to field or fertilizer treatments.

#### 3.2.3. Correlation with Crop Biomass

The relationship between crop and weed biomass varied depending on the crop system and harvest stage. At flowering, weed biomass was negatively correlated with crop biomass in high-density sole crops (C100 and Fb100) and positively correlated in low-density sole crops (C60 and Fb60), whereas no relationship was observed in intercrops (Table 6). At maturity, the relationship between crop and weed biomass was negative for all crop systems, with the strongest correlations in C100 and Fb60 and, again, the weakest in the intercrops.

### 3.3. Weed Richness

**Figure 6:**
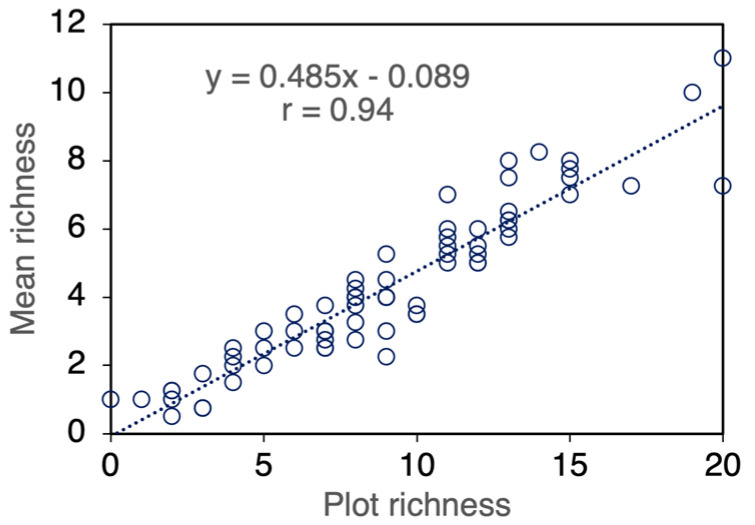
Relationship between mean richness (number of species per sampling unit) and plot richness (number of species per crop system × fertilizer plot within a field), over the entire research (*n* = 72).

**Figure 7:**
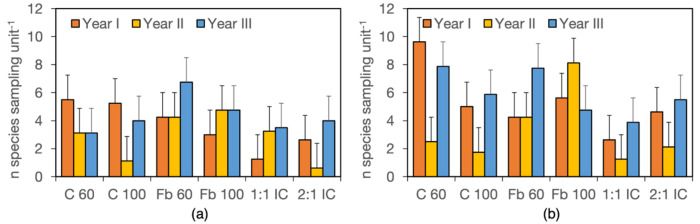
Mean richness (number of species per sampling unit), as affected by the year × field × crop system interaction over the entire research. (**a**), field A; (**b**), field B; data are means of two harvest stages, two fertiliser levels and two replicates (*n* = 8). Error bars denote HSD (*p* ≤ 0.05, Tukey’s test).

Mean richness per sampling unit was strongly correlated with plot richness (Figure 6). Both parameters were slightly higher in field B (7.8 mR and 9.6 pR) than in field A (5.3 mR and 8.5 pR), reflecting the differences in overall richness observed between the two weed communities (Table 5). Averaged over years and crop systems, NP fertilisation reduced mean richness from 4.7 to 3.8 species per sampling unit and plot richness from 9.7 to 8.3, respectively. The Year × Field × Crop System interaction revealed high variability in species number per sampling unit, ranging from 1.3 to 12.5, with no clear patterns in response to year or treatments (Figure 7a,b). However, mean richness was generally lower in intercrops and was markedly reduced in year II in cereal sole crops and in the 2:1 intercrop. Averaged across years, fields and fertiliser treatments, mean richness was highest in field bean sole crops and in C60, intermediate in C100 and the 2:1 intercrop, and lowest in the 1:1 intercrop (Table 7). Similar trends were observed for plot richness, although differences among values were slightly smaller, with richness reductions of 50.1% based on sampling units and 44.6% based on subplots.

The more than twofold higher values of plot richness compared with mean richness reflect the typically patchy distribution of weeds; however, the strong correlation between the two variables indicates that the number of species per sampling unit is a reliable proxy for overall richness.

### 3.4. Analysis of Functional Traits

#### 3.4.1. Effect of Fertiliser and Crop System

The analysis of functional traits revealed that treatments primarily affected the average frequency of functional types (AF), followed by their relative richness (RR), while the contribution of types to the weed community (TC) was the most conservative parameter. As RR showed only intermediate significance for all analysed variables, RR data are not reported. As observed for biomass, the measured variables responded more strongly to crop systems than to NP fertilisation, with no interaction between these treatments. Because, within the life form and lifespan categories, the most affected types were therophytes and annuals, respectively, only data concerning therophytes were reported and discussed to avoid redundancy.

**Figure 8:**
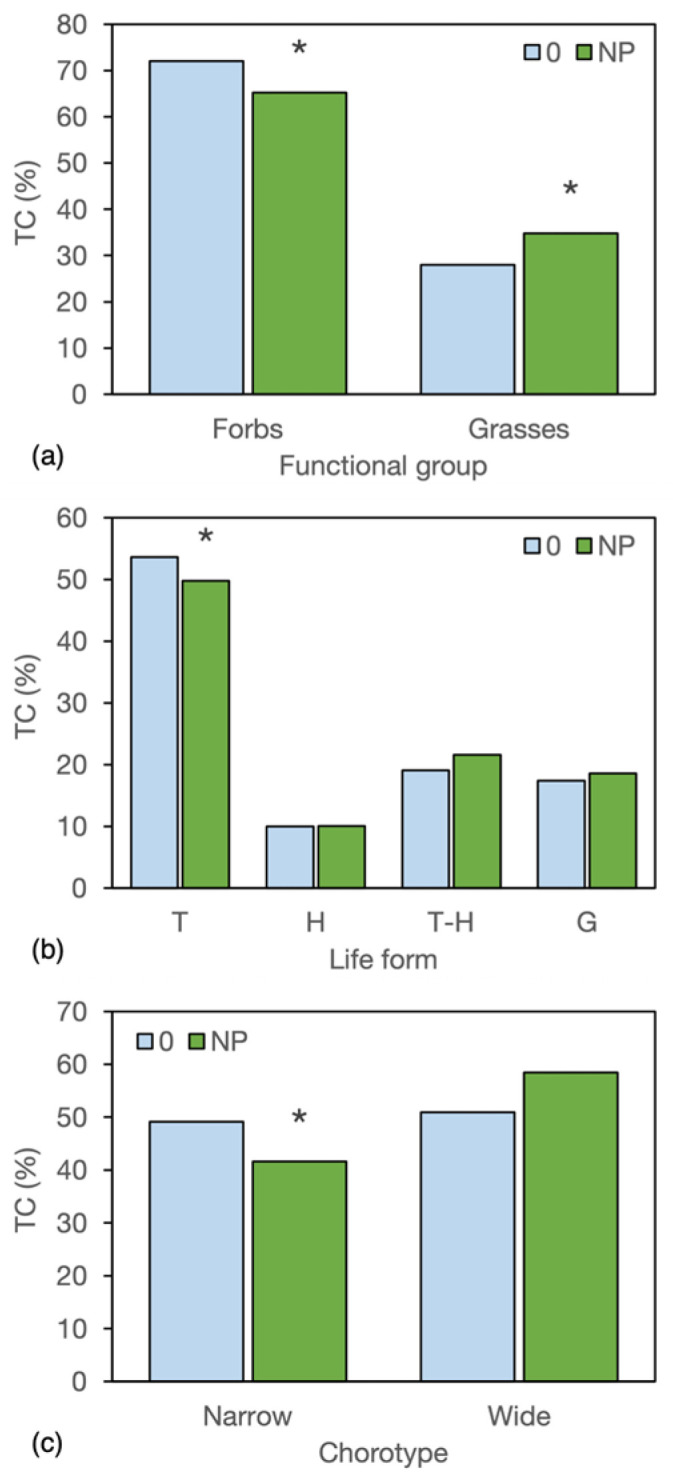
Contribution of types (%) to the weed community, as affected by the NP fertilization mean effect, over the entire research. (**a**) category functional group. (**b**) category life form. (**c**) category chorotype. Data are means of three years, two harvest stages, two fields, six crop systems, and two replicates (*n* = 144). * denotes significant differences within a type (*p* ≤ 0.05).

As expected, given the greater responsiveness of grasses to nitrogen, the contribution of forbs to the weed community decreased from 72.0% to 65.2% following NP fertilisation, whereas that of grasses increased from 28.0% to 34.8% (Figure 8a).

Fertilisation also significantly reduced the contribution of pure therophytes in favour of therophytes with some resprouting ability and geophytes (Figure 8b). In addition, NP fertilisation decreased the contribution of narrowly distributed species from 49.1% to 41.6% (Figure 8c).

Crop system affected the average frequency of at least one type within all the analysed functional categories. Specifically, significant differences among crop systems were recorded for grasses and forbs, therophytes and hemicryptophytes, early-spring, spring and late-flowering weeds, small and medium-sized weeds, and for those with both narrow and wide geographical distribution (Table 8).

Except for chorotype, the changes in the average frequency also led to changes in the contribution of types, thus affecting the functional structure of the weed community. Grasses and forbs contributed equally to the weed community in C100, whereas forbs contributed from 69 to 83% in the other crop systems, with the highest TC recorded in field bean sole crops and in the 1:1 IC (Table 9). The TC of therophytes was markedly lower in C100 and the 2:1 IC compared to C60 and the crop systems, including field bean, whereas the other life forms were not significantly affected. The contribution of early and early-spring species did not differ among crop systems, whereas that of late species decreased to the advantage of spring species in C100 and in the 2:1 IC. Approximately half of the weed species recorded in the intercrops were tall-sized, whereas this type contributed for 41.9% to the community of the high-density sole crops and one-third (C60) or less (Fb60) to the community of the low-density sole crops. The contribution of medium-sized species was not significantly affected, while that of small species was markedly lower in the 1:1 IC compared to the low-density sole crops (Table 9).

#### 3.4.2. Canonical Discriminant Analysis

**Figure 9:**
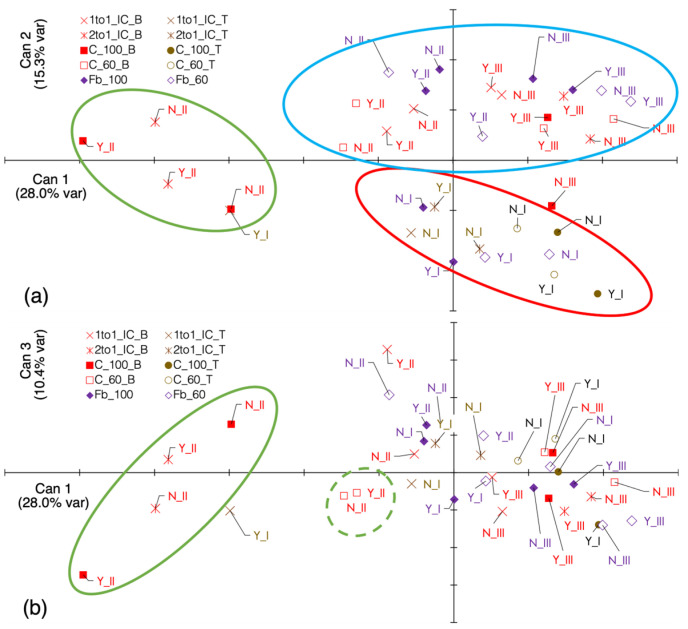
CDA ordination of 21 variables with reciprocal correlation between −0.65 and +0.65. (**a**) Distribution on the canonical axes (CA) 1 and 2. (**b**) Distribution on the CA1 and CA3. The percentages of the total variance explained by each canonical axis are shown in parentheses. Lines starting from ‘0;0’ represent the vectors of each determinant. Indicators refer to crop systems. Labels are abbreviations for fertiliser levels (Y = 0; N = NP) and years (I = year I; II = year II, III = year III). Group I (green circles); group II (blue circle); group III (red circle). In the legend: B, barley; T, triticale. Each point is the centroid mean across replicates (*n* = 8).

The CDA performed on the 21 selected variables yielded three canonical axes (CAs) with a *p*-value for the null hypothesis (H_0_) lower than 0.0041. Together, these axes explained 53.8% of the total variability of the original dataset (Figure 9). Canonical correlations associated with all remaining axes showed non-significant H_0_ *p*-values (>0.12). The weed community structure can be, therefore, summarised along three main gradients. Ten traits were identified as the main contributors of the distribution along CA1: the type contribution (TC) and average frequency of forbs; the TC of early-, early-spring- and late-flowering species; the TC of small-, medium- and tall-sized weeds; the TC of therophytes; and the TC of species with a narrow geographical distribution (Table 4; Supplementary Table S1). From the negative to the positive end, this axis represented a gradient from ruderal, short-lived, widely adapted communities to more structured communities dominated by taller forbs and functionally diverse species. The CA2 axis mainly separated cropping seasons (Figure 9a), while it was weakly linked to CA1 drivers except the TC of forbs and the TC of therophytes. These variables also influenced the distribution on the CA3 axis (Figure 9b).

The patterns of distribution in the CDA hyperspace were primarily driven by differences among cropping seasons, whereas the effect of NP fertilisation was comparatively weak and varied among years. Intercrops generally occupied intermediate positions relative to sole crops in years II and III, when barley was used, but not in year I, when a less competitive cereal (triticale) was grown and the preceding agronomic history differed between fields. Three main distribution patterns were identified in the CDA hyperspace. The strongest separation was observed for the group including both fertilised and unfertilised C100 and the 2:1 intercrop system in year II (group I), which were negatively associated with CA1. A second group (group II), comprising all crop systems in year III and part of those in year II, was located in the positive region of CA2; within this group, year II systems were mainly negatively associated with CA1, whereas year III systems were positively associated with CA1. In contrast, all crop systems in year I were negatively associated with CA2, with unfertilised treatments (N_0_ in Figure 9a) positioned slightly closer to CA1 (group III). The CA3 axis maintained the separation of group I but did not clearly discriminate between groups II and III (Figure 9b). In addition, CA3 shifted both fertilised and unfertilised C60 systems of year II towards group I.

## 4. Discussion

The number of species recorded in the two fields over the entire study period matched that of cultivated semiarid prairies [28] but was relatively high for intensive farming systems [49]. The higher richness likely reflected the absence of weed control during the research period and the reduced tillage operations carried out in both fields in previous years.

Cultural operations did not significantly reduce weed species richness. However, after three years, weed communities in the two fields showed similar richness and became more alike in species composition, indicating that continuous cultivation using the same techniques reduced differences related to field history [49,50]. Consistently, the most frequently recorded species, *Lolium multiflorum*, *Helminthotheca echioides*, *Convolvulus arvensis*, and *Chenopodium album*, are typical weeds of winter crops [51,52]. Moreover, globally widespread weeds such as *Brassica nigra*, *Lysimachia arvensis*, *Chenopodium album*, *Polygonum aviculare*, and *P. maculosa* increased in frequency over time, indicating a shift toward common ruderal species driven by cultural practices [1].

In contrast to species richness, cultural operations markedly reduced weed biomass after the first cropping season. The much higher weed biomass observed in year I compared with subsequent years may be partly due to earlier sowing [53], but mainly to a flush of germination from the seed bank triggered by intensive tillage following several years of low or no tillage [34].

Interannual variability in temperature, and especially rainfall, influenced both weed richness and biomass, with the lowest values recorded in the drier year II. Similar patterns in response to rainfall were reported for weed biomass in legume-non-legume intercrops grown in semiarid conditions [28], which supports the view that short-term environmental conditions exert a strong influence on weed dynamics [21,54]. Across all years, cereal:field bean intercropping provided greater and more stable weed suppression than sole crops throughout the crop cycle, irrespective of cereal:field bean row ratio or NP fertiliser distribution. The weed suppression of sole crops was generally greater in cereals than in field beans but varied widely among years, growth stages, and crop densities. The ranking of weed suppression observed in this study is robust, as it was consistent despite marked differences in climate, field history, sowing dates, weed biomass between year I and later years, and the replacement of triticale with barley. Similar rankings have been reported for chickpea:wheat and pea:barley intercrops [14,55].

Overall, high-density sole crops (Fb100 and C100) provided better weed control than low-density sole crops (Fb60 and C60), although effectiveness varied among fields and years [13]. In all years, C100 showed weed suppression comparable to intercrops before flowering, but not thereafter; at maturity, weed biomass in C100 was approximately double that of intercrops, despite similar row spacing and equal or lower seed density [21]. These patterns suggest that in intercrops, weed control benefits from the combined competitive effects of cereal and field bean crops and from their differing critical periods for weed development during the crop cycle [29]. A wide range of organic cereal–legume intercrops examined across Western Europe confirmed our findings, suggesting that co-cultured crops probably diminish the competitive ability of weeds through a more complete soil N exploitation [30]. Cereals are generally more competitive than weeds during early growth stages due to the rapid development of a dense root system [14], whereas field bean exerts strong shading effects and increases competition for mineral nitrogen after flowering [52,56].

The positive relationships between crop and weed biomass recorded at flowering in C60 and Fb60 confirm that environmental conditions and nutrient availability are the primary drivers of biomass accumulation in both weeds and crops in low-density systems at early growth stages, regardless of whether crops are legumes or cereals. In contrast, weed growth was negatively related to crop biomass throughout the crop cycle in high-density sole crops [20,31,55]. The weak relationship between crop and weed biomass observed in intercrops supports the hypothesis that crop diversity contributes to weed suppression [16,19].

Crop systems influenced the partitioning of forbs and grasses within weed biomass, although the high variability among years and fields suggests a strong influence of the patchy weed seed bank distribution [18]. Nevertheless, when averaged across years, fields, and fertiliser treatments, forbs contributed less to weed biomass and richness in high-density cereal crops than in other systems, potentially reducing floral resources for pollinators [1].

Over the three-year period, changes in weed functional composition were less pronounced than changes in biomass, confirming that abundance responds more rapidly than composition and that longer periods may be required to detect compositional shifts [34,36]. Strong weed biomass suppression was associated with reduced mean species richness in both high-density cereal sole crops and intercrops; thus, our results do not support the hypothesis that increased crop diversity enhances weed diversity [57]. Due to the spatial heterogeneity of the weed seed bank, a reduction in mean richness does not necessarily imply a loss of weed biodiversity [9,21]. However, we observed similar patterns of decline for the plot richness, and Fernandez et al. [28] reported a lower seed density in cereals grown after lentil-mustard and pea-oat intercrops. We cannot, therefore, exclude the possibility that continuous intercropping may progressively lead to a simplification of the weed community, particularly under the 1:1 row ratio.

Functional analysis showed that intercrops similarly reduced the average frequency and relative richness of most functional types. As a result, changes in their proportional contributions were limited. The contribution of forbs in intercrops resembled that observed in the less competitive cropping systems (C60 and Fb sole crops) and was higher than in C100. Only tall weed species showed a higher contribution in intercrops compared with all other cropping systems, reflecting stronger competition against small- and medium-sized weeds. This pattern is consistent with the combined effects of high crop density and differential resource use by cereal and legume crops throughout the crop cycle [1,15].

As reported by Poggio [21], continuous intercropping altered the contribution of annual species (therophytes) and flowering phenology types, with different responses depending on the cereal:legume row proportion. In the 1:1 intercrop, the contribution of pure annual therophytes and late-flowering weeds was similar to that observed in field bean sole crops and low-density cereals, whereas in the 2:1 intercrop it resembled that of C100. This suggests that cereal proportion, rather than plant density alone, drove the decline in pure therophytes and shifted flowering phenology from late to spring-flowering species.

The distribution of NP fertiliser had limited effects on weed biomass, indicating a more efficient use of nutrients by both the cereal and legume crops than by weeds [58]. Differences from studies on pea–barley intercrops [55] may reflect the greater nitrogen responsiveness of field bean [59]. In year I, however, a positive weed response to NP fertilisation was detected in the low-density crops (C60 and Fb60), suggesting that nutrient inputs exceeded crop demand, leaving nitrogen and phosphorus available for weed growth. Notably, weed biomass was never affected by the interaction between field and fertiliser, indicating that weed growth responded primarily to nutrient availability, irrespective of field history or weed community composition. In year I, NP fertilisation also reduced forb contribution in field B, which may be explained by the dominance of the tall, highly nitrogen-responsive grass *Lolium multiflorum* in the weed community in that field [60].

Across all years and fields, NP fertilisation altered weed community composition by reducing the contribution of forbs and therophytes. It also reduced the contribution of narrowly distributed species, which, together with the slight decline in species richness per sampling unit, may negatively affect weed biodiversity at both field and local scales [49,58].

The CDA revealed that weed community structure was primarily driven by cropping season; weed communities were more similar across crop systems in the years with more favourable environmental conditions [24,54], while fertilisation had only a minor and inconsistent influence on community structure. The greatest separation occurred for C100 and the 2:1 intercrop in year II, corresponding to cropping systems with a high barley proportion under drier and colder conditions in which crops exerted a great dominance over weeds. You et al. [61] reported that nitrogen fertilisation, in addition to rainfall and temperature, influenced the functional composition of grasslands. In the present study, however, fertiliser effects on weeds were not clearly detectable, likely because they were masked by the stronger effects of nitrogen on crops, particularly those with a high cereal proportion.

## 5. Conclusions

The present study demonstrated that intercropping field beans with cereals in the same plot for three consecutive years suppressed weed biomass much more effectively than sole crops throughout the entire crop cycle, with lower interannual variability. Intercropping may therefore represent an economically and environmentally sustainable strategy for reducing the negative impacts of weeds on crop production.

The strong suppression of weed growth was associated with a slight reduction in species richness and an increased contribution of tall weed species, which could, over time, favour the dominance of one or a few more aggressive species. Except for plant size types, intercropping did not substantially alter the functional composition of the weed community: the 1:1 intercrop resembled the field bean sole crops, whereas the 2:1 intercrop was more similar to the cereal sole crops. From a methodological perspective, our findings support the hypothesis that functional trait analysis can overcome differences in species composition and that the average frequency of functional types, being more sensitive to change than their proportional contribution, may serve as an early indicator of shifts in weed community structure.

However, the canonical discriminant analysis indicated that year-to-year variation exerted a stronger influence on the functional composition of weed communities than NP fertilisation or crop system. This suggests that longer-term experiments are required to disentangle treatment effects from the confounding influences of inherent spatial heterogeneity in weed seed banks and interannual climatic variability.

## Figures and Tables

**Table 1:** Row spacing adopted for the crop systems over the entire research, and seed density and field bean proportion in seed adopted in year I and in years II and III. In the cereal (C) and field bean (Fb) sole crops, acronyms indicate the percentage proportion to the highest density (100); in the intercrops (IC), acronyms indicate the C:Fb row ratio.

			Year I		Years II and III	
Crop System	Acronym	Row Spacing (cm)	Seed Density (n m^−2^)	Fb Seed Proportion	Seed Density (n m^−2^)	Fb Seed Proportion
Sole crop	C100	15	400	0.0	200	0.0
	C60	23	240	0.0	120	0.0
	Fb100	30	100	1.0	100	1.0
	Fb60	45	60	1.0	60	1.0
Intercrop	1:1 IC	15	300	0.3	200	0.5
	2:1 IC	15	300	0.2	180	0.3

**Table 2:** Main physical and chemical soil properties of the 0–30 cm soil profile measured in the two fields at the beginning of the experiment (October 2017).

Soil Properties	Unit	Field A	Field B
Sand (50–2000 μm)	%	45.3	46.1
Silt (2–50 μm)	%	44.6	42.5
Clay (<2 μm)	%	10.1	11.4
Soil Organic Matter	%	2.64	3.05
pH	-	8.3	8.2
Total N	g kg^−1^	1.33	1.87
Available P	mg kg^−1^	18.9	12.3
Exchangeable K	mg kg^−1^	201.6	266.6
Total limestone	%	8.09	6.35
Electrical Conductivity	dS m^−1^	0.24	0.23
Cation Exchange Capacity	meq 100 g^−1^	10.8	11.3
Total Zn	mg kg^−1^	73.6	70.8
Total Fe	g kg^−1^	30.0	28.6
Water holding capacity	%	15	16
Water saturation	%	48	46
Bulk density	g cm^−3^	1.4	1.3

**Table 3:** Sowing and harvesting dates in the three cropping seasons.

Cropping Year	Sowing	Vegetative	Flowering	Maturity
Year I	26 October 2017	23 February 2018	5 May 2018	2 July 2018
Year II	11 December 2018	-	11 May 2019	29 June 2019
Year III	12 February 2020	-	1 June 2020	30 June 2020

**Table 4:** Number of species recorded each year in field A (A) and in field B (B), and list of the weed species recorded over the research, with their frequency (%) in the sampling units collected each year in one field (*n* = 48, resulting from two harvest dates, two fertilizer treatments, six crop systems, and two replicates). “-” indicates non-occurrence (i.e., species was not present).

N°	Species	Year I		Year II		Year III	
		A	B	A	B	A	B
	Number of species	24	33	23	16	27	27
1	*Cyperus rotundus*	10.4	39.6	-	-	10.4	8.3
2	*Juncus bufonius*	4.2	4.2	-	-	-	-
3	*Alopecurus myosuroides*	31.3	-	39.6	-	25.0	2.1
4	*Avena barbata*	16.7	2.1	2.1	-	4.2	-
5	*Cynodon dactylon*	8.3	-	-	-	2.1	-
6	*Lolium multiflorum*.	27.1	79.2	20.8	79.2	41.7	60.4
7	*Phalaris brachystachys*	-	6.3	-	4.2	-	2.1
8	*Phalaris paradoxa*	2.1	2.1	-	2.1	2.1	2.1
9	*Poa trivialis*	6.3	25.0	10.4	10.4	2.1	12.5
10	*Ammi majus*	12.5	6.3	-	-	-	-
11	*Daucus carota*	-	20.8	-	2.1	2.1	4.2
12	*Anthemis arvensis*	-	6.3	-	-	-	18.8
13	*Coleostephus myconis*	-	4.2	-	6.3	-	6.3
14	*Helminthotheca echioides*	68.8	56.3	20.8	14.6	41.7	60.4
15	*Sonchus arvensis*	8.3	6.3	8.3	2.1	4.2	2.1
16	*Brassica nigra*	-	4.2	-	2.1	4.2	10.4
17	*Capsella bursa-pastoris*	-	-	4.2	-	-	-
18	*Rapistrum rugosum*	2.1	-	-	-	8.3	-
19	*Cerastium glomeratum*	-	18.8	4.2	-	-	-
20	*Stellaria media*	-	2.1	-	-	-	-
21	*Beta vulgaris*	12.5	-	2.1	-	8.3	-
22	*Chenopodium album*	-	-	31.3	16.7	33.3	75.0
23	*Calystegia sepium*	2.1	-	4.2	-	10.4	-
24	*Convolvulus arvensis*	45.8	52.1	12.5	35.4	37.5	31.3
25	*Equisetum ramosissimum*	20.8	-	10.4	-	4.2	-
26	*Equisetum arvense*	4.2	6.3	18.8	10.4	10.4	14.6
27	*Centaurium erythraea*	-	8.3	-	-	-	-
28	*Oxalis corniculata*	-	-	-	-	14.6	-
29	*Papaver rhoeas*	12.5	16.7	25.0	18.8	20.8	56.3
30	*Plantago major*	8.3	2.1	16.7	-	33.3	12.5
31	*Veronica persica*	-	8.3	4.2	12.5	10.4	22.9
32	*Veronica serpyllifolia*	-	-	6.3	-	20.8	2.1
33	*Polygonum aviculare*	2.1	22.9	39.6	20.8	52.1	43.8
34	*Polygonum maculosa*	-	-	-	-	-	45.8
35	*Rumex crispus*	-	-	2.1	-	-	-
36	*Lysimachia arvensis*	31.3	60.4	37.5	20.8	64.6	91.7
37	*Adonis annua*	-	-	2.1	-	-	-
38	*Ranunculus velutinus*	-	-	-	-	2.1	-
39	*Reseda lutea*	2.1	-	-	-	-	2.1
40	*Solanum nigrum*	-	2.1	-	-	-	-
41	*Verbena officinalis*	10.4	4.2	8.3	-	20.8	-
42	Unknown	-	10.4	-	-	-	-
43	*Sulla coronaria*	-	4.2	-	-	-	2.1
44	*Medicago lupulina*	-	6.3	-	-	-	6.3
45	*Medicago polymorpha*	-	4.2	-	-	-	10.4
46	*Trifolium campestre*	10.4	8.3	-	-	-	-
47	*Trifolium repens*	-	27.1	-	-	-	4.2
48	*Trifolium squarrosum*	-	4.2	-	-	-	-

**Table 5:** Number of weed species recorded in the fields A and B over three years (Total), and number and proportion in the weed community of species belonging to the categories: functional group (use of resources), life span (duration of life cycle), and chorotype (geographical distribution).

Functional Category	Type	Acronym	Total		Field A		Field B	
			*n*	%	*n*	%	*n*	%
	Total	N/A *	48		35		38	
Functional group	Grasses	Gr	9	18.8	8	22.9	8	21.0
	Forbs	Fo	33	68.8	26	74.2	24	63.2
	Legumes	Lg	6	12.4	1	2.9	6	15.8
Life span	Annuals	A	23	47.9	16	45.7	21	55.3
	Others	B, P, AP	25	52.1	19	54.3	17	44.7
Chorotype	Narrow	N	23	47.9	17	48.6	17	44.7
	Wide	W	25	52.1	18	51.4	21	55.3

* N/A indicates not applicable.

**Table 6:** Crop and weed biomass (g DW m^−2^) and correlation coefficient (r) of their relationship at flowering and maturity. Data refer to three years, two fields, two fertilizer treatments, and two replicates (*n* = 24). Critical r value 0.359.

	Flowering			Maturity		
CROP SYSTEM	Crop	Weed	r	Crop	Weed	r
C 60	785.8	47.8	+0.39	907.3	156.2	−0.16
C 100	1017.7	11.6	−0.48	1129.1	61.3	−0.43
Fb 60	760.1	91.8	+0.46	884.8	237.3	−0.41
Fb 100	972.5	31.6	−0.35	1012.8	143.5	−0.31
1:1 IC	1116.4	14.5	−0.17	1265.9	22.1	−0.08
2:1 IC	1065.6	11.8	−0.09	1194.8	23.7	−0.08

**Table 7:** Mean richness (*n* of species per sampling unit) and plot richness (*n* of species recorded per subplot over all harvest within a year), as affected by the crop system mean effect. Mean richness *n* = 48; plot richness *n* = 12. Within a row, means followed by different letters indicate statistical difference at *p* ≤ 0.05, Tukey’s test.

	C60	C100	Fb60	Fb100	1:1 IC	2:1 IC
	*n* of species					
Mean richness	5.3 a	3.8 ab	5.3 a	5.2 a	2.6 b	3.3 ab
Plot richness	10.8 a	8.5 b	10.8 a	10.8 a	6.0 c	7.2 bc

**Table 8:** Average frequency (%) of types within functional categories, as affected by the crop system mean effect. Data are means of three years, two fields, two fertiliser levels, and four replicates (*n* = 48). Means followed by different letters within a row indicate statistical difference among types at *p* ≤ 0.05, Tukey’s test.

Crop Systems	C60	C100	Fb60	Fb100	1:1 IC	2:1 IC
Category/Type						
Functional group	Average frequency (%)					
Grasses	20.5 a	20.4 a	21.0 a	17.8 a	13.4 b	12.2 b
Forbs	21.1 a	13.3 b	25.4 a	22.4 a	9.9 b	13.4 b
Life form						
Therophytes	22.6 a	17.0 b	29.6 a	25.5 a	12.9 b	13.2 b
Hemicryptophytes	13.3 a	5.4 b	14.0 a	11.4 a	3.9 b	3.7 b
Thero-Hemicrypt.	21.8 a	21.8 a	23.9 a	22.1 a	14.2 a	18.3 a
Geophytes	22.8 a	18.8 a	19.2 a	23.5 a	10.9 a	23.4 a
Earliness						
Early	12.5 a	9.5 a	14.4 a	8.5 a	5.0 a	5.1 a
Early-Spring	21.8 ab	13.9 b	28.3 a	18.9 ab	9.6 b	9.7 b
Spring	22.2 a	17.9 ab	21.6 a	24.1 a	13.6 b	18.9 ab
Late	26.2 a	16.1 b	30.7 a	28.9 a	12.7 b	15.1 b
Size						
Small	23.6 ab	16.4 bc	29.5 a	21.8 b	12.2 c	10.7 c
Medium	21.0 ab	14.2 bc	26.8 a	18.6 b	7.4 c	10.3 c
Tall	16.5 a	13.2 a	17.2 a	23.0 a	13.7 a	17.1 a
Chorotype						
Narrow	16.6 ab	15.0 b	21.1 a	17.8 ab	8.5 c	10.3 bc
Wide	23.4 a	14.8 b	25.4 a	23.6 a	12.1 b	14.9 b

**Table 9:** Contribution of types (%), as affected by the crop system mean effect. Data are means of three years, two fields, two fertiliser levels, and four replicates (*n* = 48). Means followed by different letters within a row indicate statistical difference among types at *p* ≤ 0.05, Tukey’s test.

Crop Systems	C60	C100	Fb60	Fb100	1:1 IC	2:1 IC
Category/Type						
Functional group	Type contribution (%)					
Grasses	28.3 b	49.2 a	18.8 c	16.8 c	21.1 c	31.1 b
Forbs	71.7 b	50.8 c	81.2 a	83.2 a	78.9 a	68.9 b
Life form						
Therophytes	51.6 abc	47.0 bc	58.7 a	55.6 ab	54.5 ab	41.8 c
Hemicryptophytes	13.5 a	5.6 a	12.6 a	11.1 a	8.4 a	9.2 a
Thero-Hemicrypt.	17.8 a	28.5 a	16.3 a	15.6 a	19.8 a	24.3 a
Geophytes	17.2 a	18.9 a	12.3 a	17.7 a	17.4 a	24.7 a
Earliness						
Early	5.4 a	7.1 a	7.2 a	6.0 a	7.4 a	5.3 a
Early-Spring	29.8 a	26.5 a	33.7 a	25.5 a	23.2 a	20.7 a
Spring	35.4 bc	43.7 ab	27.6 c	35.5 bc	39.1 abc	49.6 a
Late	29.4 ab	22.6 c	31.5 a	33.0 a	30.3 a	24.5 bc
Size						
Small	31.1 a	23.2 ab	30.1 a	25.2 ab	26.5 ab	19.5 b
Medium	34.2 a	34.9 a	42.8 a	33.0 a	22.3 a	29.9 a
Tall	34.8 bc	41.9 ab	27.1 c	41.9 ab	51.3 a	50.6 a
Chorotype						
Narrow	40.5 a	55.7 a	48.5 a	43.1 a	41.9 a	46.7 a
Wide	59.5 a	44.3 a	51.5 a	56.9 a	58.2 a	53.3 a

## Data Availability

The original contributions presented in this study are included in the article/Supplementary Material. Further inquiries can be directed to the corresponding author.

## Supplementary Materials

The following supporting information can be downloaded at: https://www.mdpi.com/article/10.3390/biology15030239/s1.

## Abbreviations

The following abbreviations are used in this manuscript:

AF	Average frequency
CA	Canonical Axsis
CDA	Canonical Discriminant Analysis
C	Cereal
C100	High density cereal sole crop
C60	Low density cereal sole crop
Fb	Field bean
Fb100	High density field bean sole crop
Fb60	Low density field bean sole crop
IC	Intercrop
1:1 IC	Cereal:field bean 1:1 row ratio intercrop
2:1 IC	Cereal:field bean 2:1 row ratio intercrop
mR	Mean richness
pR	Plot richness
RR	Relative richness
SC	Sole crop
TC	Type contribution

## Appendix A

Details on functional categories (Table A1) and functional classification of the weed species recorded over the research (Table A2).

**Table A1:** Description of functional trait categories and types associated with weed species.

Functional Category	Type	Acronym	Plant Trait
Functional group (use of resources)	Grasses	Gr	Extended fibrous root system, tillering ability, anemogamous (*Poaceae* and *Cyperaceae*)
	Forbs	Fo	Diverse shoot forms, most entomogamous (Dicotyledones except *Fabaceae*)
	Legumes	Lg	N_2_-fixing *Fabaceae*
Life form (Raunkiaer’s survival strategy)	Therophyte	T	Only seeds survive the adverse season
	Hemicryptophyte	H	Quiescent buds at the base of shoots
	Geophyte	G	Quiescent buds on belowground organs
		TH	Therophytes with some resprouting ability
Life span (duration of life cycle)	Annuals	A	One growth season
	Biennials	B	Two growth seasons
	Perennials	P	More than two growth seasons
		AP	Annuals, optionally able to last more seasons
Earliness (flowering relative to crops)	Early	E	Flowering and maturing seeds before strong shading from the crop canopy
	Spring	S	Flowering and maturing seeds before forage harvest
	Late	L	Flowering and maturing seeds similar to crops
Size (plant height relative to crops)	Small	s	<20 cm, completely shaded by crops for the entire growth cycle
	Medium	m	20–100 cm, shaded by crops for at least part of the growth cycle
	Tall	t	≥100 cm, able to compete with crops for light
Chorotype (range of geographic and ecological distribution)	Narrow	N	Species with a given geographical distribution
	Wide	W	Cosmopolite and Subcosmopolite species

**Table A2:** Botanical classification and characterisation according to plant functional types and chorotype of the weed species recorded over the three-year experiment. Refer to Table A1 for the acronyms and the description of types. In brackets, the flowering month and the size class observed in the field.

Species	Family	Funct. Group	Life Form	Life Span	Earliness (Month)	Size Class	Chorotype
*Cyperus rotundus* L.	Cyperaceae	Gr	G	P	L (6–11)	m (s)	Subcosm.
*Juncus bufonius* L.	Juncaceae	Gr	T	A	S (5–9)	s	Cosmop.
*Alopecurus myosuroides* Huds.	Poaceae	Gr	T	A	E-S (4–6)	m	Subcosm.
*Avena barbata* Pott ex Link	Poaceae	Gr	T	A	E-S (4–6)	t	Euri-Medit.-T
*Cynodon dactylon* (L.) Pers.	Poaceae	Gr	G	P	L (6–9)	m (s)	Cosmop.
*Lolium multiflorum* Lam.	Poaceae	Gr	TH	AP	S (5–7)	t	Euri-Medit.
*Phalaris brachystachys* Link.	Poaceae	Gr	T	A	E-S (4–6)	t	Steno-Medit.
*Phalaris paradoxa* l.	Poaceae	Gr	T	A	E-S (4–5)	t	Steno-Medit.
*Poa trivialis* L.	Poaceae	Gr	H	P	S (5–9)	m	Eurasiat.
*Ammi majus* L-	Apiaceae	Fo	T	A	S (5–7)	t	Euri-Medit.
cfr. *Daucus carota* L.	Apiaceae	Fo	TH	AB	L (4–10)	m	Subcosm.
*Anthemis arvensis* L.	Asteraceae	Fo	TH	AP	E-S (4–6)	m	Subcosm.
*Coleostephus myconis* (L.) Cass. ex Rchb	Asteraceae	Fo	T	A	E-S (4–7)	m	Steno-Medit
*Helminthotheca echioides* (L.) Holub	Asteraceae	Fo	T	A	L (6–8)	m	Euri-Medit.
*Sonchus arvensis* L.	Asteraceae	Fo	H	P	L (6–9)	t	Subcosm.
*Brassica* cfr. *nigra* (L.) W.D.J.Koch	Brassicaceae	Fo	T	A	E (2–5)	t	Medit.
*Capsella bursa-pastoris* (L.) Medik.	Brassicaceae	Fo	H	B	E (1–12)	s	Cosmop.
*Rapistrum rugosum* (L.) All.	Brassicaceae	Fo	T	A	S (5–7)	t	Euri-Medit.
*Cerastium glomeratum* Thuill.	Caryophyllaceae	Fo	T	A	E (1–12)	s	Subcosm.
*Stellaria media* (L.) Vill.	Caryophyllaceae	Fo	TH	A-B	E (1–12)	s	Subcosm.
*Beta vulgaris* L.	Chenopodiaceae	Fo	TH	A-B	L (6–8)	t	Euri-Medit.
*Chenopodium album* L.	Chenopodiaceae	Fo	T	A	L (6–9)	t	Subcosm.
*Calystegia sepium* (L.) R. Br.	Convolvulaceae	Fo	H	P	S (5–9)	t	Paleotemp.
*Convolvulus arvensis* L.	Convolvulaceae	Fo	G	P	S (4–10)	t	Cosmop.
*Equisetum arvense* L.	Equisetaceae	Fo	G	P	S (5–8)	m	Circumb.
*Equisetum ramosissimum* Desf.	Equisetaceae	Fo	G	P	S (5–9)	m	Circumb.
*Centaurium erythraea* Rafn	Gentianaceae	Fo	TH	A-B	S (5–9)	s	Paleotemp.
*Oxalis corniculata* L.	Oxalidaceae	Fo	H	P	E-S (4–6)	s	Cosmop.
*Papaver rhoeas* L.	Papaveraceae	Fo	T	A	E-S (4–6)	m	E-Medit.
*Plantago major* L.	Plantaginaceae	Fo	H	P	E-S (4–9)	s	Subcosm.
*Veronica persica* Poir.	Plantaginaceae	Fo	T	A	E (1–12)	s	A-Subcosm.
*Veronica serpyllifolia* L.	Plantaginaceae	Fo	H	P	S (5–10)	s	Subcosm.
*Polygonum aviculare* L.	Polygonaceae	Fo	T	A	L (6–10)	s	Cosmop.
*Polygonum maculosa* Gray	Polygonaceae	Fo	T	A	L (7–10)	m	Subcosm.
*Rumex crispus* L.	Polygonaceae	Fo	H	P	S (5–7)	t	Subcosm.
*Lysimachia arvensis* (L.) U.Manns & Anderb.	Primulaceae	Fo	T	A	E-S (4–10)	s	Subcosm.
*Adonis annua* L.	Ranunculaceae	Fo	T	A	E (3–6)	m	Euri-Medit.
*Ranunculus velutinus* Ten.	Ranunculaceae	Fo	H	P	E-S (4–6)	m (t)	N-Medit.
*Reseda lutea* L.	Resedaceae	Fo	T,H	A-P	S (5–9)	m	Europ.
*Solanum nigrum* L.	Solanaceae	Fo	T	A	E (3–12)	t	Cosmop
*Verbena officinalis* L.	Verbenaceae	Fo	H	P	E (3–10)	m	Cosmop.
Unknown		Fo					
*Sulla coronaria* (L.) Medik.	Fabaceae	Lg	H	P	E-S (4–5)	t	W-Medit.
*Medicago lupulina* L.	Fabaceae	Lg	T,H	A-P	E-S (4–7)	s	Paleotemp.
*Medicago polymorpha* L.	Fabaceae	Lg	T	A	E (3–5)	m	Subcosm.
*Trifolium campestre* Schreb.	Fabaceae	Lg	T	A	S (4–8)	s	W-Paleot.
*Trifolium repens* L.	Fabaceae	Lg	H	P	E-S (4–10)	s	Subcosm.
*Trifolium squarrosum* L.	Fabaceae	Lg	T	A	E-S (4–6)	m	Euri-Medit.
