# Supplementary material for: Three-Year Cereal: Field Bean Intercropping Greatly Reduced Weed Abundance with Small Changes in Functional Composition
Source: Biology (Basel). 2026 Jan 28;15(3):239. doi: 10.3390/biology15030239 (PMC12897063; doi:10.3390/biology15030239)
Supplement: Supplementary file 1 [file biology-15-00239-s001.zip › Tables_S1-S4.pdf]

Table S1. Results of ANOVA for weed biomass in year I.

individuali in verde.

▸ Stime dei parametri

▾ Test degli effetti

Origine	Nparm	DF	Somma dei quadrati	Rapporto F	Prob > F
Harvest	2	2	33.217397	205.1546	<.0001*
Campo	1	1	0.606858	7.4961	0.0078*
Harvest*Campo	2	2	1.027389	6.3453	0.0029*
Fert	1	1	0.022544	0.2785	0.5993
Harvest*Fert	2	2	0.701064	4.3299	0.0168*
Campo*Fert	1	1	0.011280	0.1393	0.7100
Harvest*Campo*Fert	2	2	0.020604	0.1273	0.8807
CropSyst	5	5	21.243543	52.4811	<.0001*
Harvest*CropSyst	10	10	7.245099	8.9493	<.0001*
Campo*CropSyst	5	5	2.935890	7.2530	<.0001*
Harvest*Campo*CropSyst	10	10	0.788829	0.9744	0.4732
Fert*CropSyst	5	5	1.691535	4.1788	0.0022*
Harvest*Fert*CropSyst	10	10	2.149117	2.6546	0.0081*
Campo*Fert*CropSyst	5	5	0.906147	2.2386	0.0595
Harvest*Campo*Fert*CropSyst	10	10	1.415199	1.7481	0.0864

Table S2. Results of ANOVA for forb proportion in the weed biomass in year I.

▾ Risposta Weed DW LOG(x+1)

▾ Test degli effetti

Origine	Nparm	DF	Somma dei quadrati	Rapporto F	Prob > F
Harvest stage	1	1	0.220052	1.3526	0.2468
Field	1	1	1.782552	10.9567	0.0012*
Harvest stage*Field	1	1	0.341719	2.1004	0.1494
Fert	1	1	0.022969	0.1412	0.7077
Harvest stage*Fert	1	1	0.596302	3.6652	0.0575
Field*Fert	1	1	0.169219	1.0401	0.3095
Harvest stage*Field*Fert	1	1	0.412552	2.5358	0.1135
Crop	5	5	16.489635	20.2711	<.0001*
Harvest stage*Crop	5	5	4.212135	5.1781	0.0002*
Field*Crop	5	5	6.688385	8.2222	<.0001*
Harvest stage*Field*Crop	5	5	0.614219	0.7551	0.5837
Fert*Crop	5	5	1.165469	1.4327	0.2160
Harvest stage*Fert*Crop	5	5	0.869635	1.0691	0.3801
Field*Fert*Crop	5	5	2.317969	2.8495	0.0174*
Harvest stage*Field*Fert*Crop	5	5	0.422135	0.5189	0.7616

Table S3. Results of ANOVA for weed biomass in years II and III.

Modello misto lineare generalizzato per Herb arcsin

Normale

Stime di parametri con effetti fissi

Test sugli effetti fissi

Origine	Nparm	Num DF	Den DF	Rapporto F	Prob > F
Harvest	1	1	48.0	6.7290022	0.0125*
Field	1	1	48.0	23.468377	<.0001*
Harvest*Field	1	1	48.0	3.1803869	0.0809
Fertilizer	1	1	48.0	2.7678857	0.1027
Harvest*Fertilizer	1	1	48.0	0.1606673	0.6903
Field*Fertilizer	1	1	48.0	4.3501853	0.0423*
Harvest*Field*Fertilizer	1	1	48.0	0.3529568	0.5552
Crop System	5	5	48.0	6.6053983	<.0001*
Harvest*Crop System	5	5	48.0	2.090418	0.0828
Field*Crop System	5	5	48.0	4.7000554	0.0014*
Harvest*Field*Crop System	5	5	48.0	0.4309523	0.8248
Fertilizer*Crop System	5	5	48.0	1.8642749	0.1183
Harvest*Fertilizer*Crop System	5	5	48.0	1.0301866	0.4107
Field*Fertilizer*Crop System	5	5	48.0	1.0892155	0.3785
Harvest*Field*Fertilizer*Crop System	5	5	48.0	1.0902294	0.3779

Table S4. Results of ANOVA for forb proportion in the weed biomass in years II and III.

Test sugli effetti fissi

Origine	Nparm	Num DF	Den DF	Rapporto F	Prob > F
Harvest stage	1	1	143.0	1.1154244	0.2927
Field	1	1	143.0	1.7115679	0.1929
Harvest stage*Field	1	1	143.0	3.5326934	0.0622
Fert	1	1	143.0	0.1432456	0.7056
Harvest stage*Fert	1	1	143.0	1.2790853	0.2600
Field*Fert	1	1	143.0	0.081032	0.7763
Harvest stage*Field*Fert	1	1	143.0	0.5568237	0.4568
Crop	5	5	143.0	3.9372119	0.0022*
Harvest stage*Crop	5	5	143.0	1.4536796	0.2088
Field*Crop	5	5	143.0	8.325111	<.0001*
Harvest stage*Field*Crop	5	5	143.0	0.5818783	0.7138
Fert*Crop	5	5	143.0	2.5051349	0.0330*
Harvest stage*Fert*Crop	5	5	143.0	1.0502125	0.3907
Field*Fert*Crop	5	5	143.0	0.9112298	0.4756
Harvest stage*Field*Fert*Crop	5	5	143.0	1.9577708	0.0885
